# Prevalence and incidence of COPD in smokers and non-smokers: the Rotterdam Study

**DOI:** 10.1007/s10654-016-0132-z

**Published:** 2016-03-05

**Authors:** Natalie Terzikhan, Katia M. C. Verhamme, Albert Hofman, Bruno H. Stricker, Guy G. Brusselle, Lies Lahousse

**Affiliations:** 1 Department of Respiratory Medicine, Ghent University Hospital, De Pintelaan 185, 9000 Ghent, Belgium; 2 Department of Epidemiology, Erasmus Medical Center, PO Box 2040, 3000 CA Rotterdam, The Netherlands; 3 Department of Internal Medicine, Erasmus Medical Center, PO Box 2040, 3000 CA Rotterdam, The Netherlands; 4 Inspectorate of Healthcare, Utrecht, The Netherlands; 5 Department of Respiratory Medicine, Erasmus Medical Center, PO Box 2040, 3000 CA Rotterdam, The Netherlands; 6 Department of Medical Informatics, Erasmus University Medical Center, PO Box 2040, 3000 CA Rotterdam, The Netherlands

**Keywords:** COPD, GOLD, LLN, Prevalence, Incidence, The Rotterdam Study

## Abstract

COPD is the third leading cause of death in the world and its global burden is predicted to increase further. Even though the prevalence of COPD is well studied, only few studies examined the incidence of COPD in a prospective and standardized manner. In a prospective population-based cohort study (Rotterdam Study) enrolling subjects aged ≥45, COPD was diagnosed based on a pre-bronchodilator obstructive spirometry (FEV_1_/FVC < 0.70). In absence of an interpretable spirometry within the Rotterdam Study, cases were defined as having COPD diagnosed by a physician on the basis of clinical presentation and obstructive lung function measured by the general practitioner or respiratory physician. Incidence rates were calculated by dividing the number of incident cases by the total number of person years of subjects at risk. In this cohort of 14,619 participants, 1993 subjects with COPD were identified of whom 689 as prevalent ones and 1304 cases as incident ones. The overall incidence rate (IR) of COPD was 8.9/1000 person-years (PY); 95 % Confidence Interval (CI) 8.4–9.4. The IR was higher in males and in smokers. The proportion of female COPD participants without a history of smoking was 27.2 %, while this proportion was 7.3 % in males. The prevalence of COPD in the Rotterdam Study is 4.7 % and the overall incidence is approximately 9/1000 PY, with a higher incidence in males and in smokers. The proportion of never-smokers among female COPD cases is substantial.

**Electronic supplementary material:**

The online version of this article (doi:10.1007/s10654-016-0132-z) contains supplementary material, which is available to authorized users.

## Introduction

Worldwide, Chronic Obstructive Pulmonary Disease (COPD) is the third leading cause of death [1]. COPD is characterized by persistent airflow limitation that is typically progressive and associated with an enhanced chronic inflammatory response in the airways and lung tissue to harmful particles or gases [2]. The chronic airflow limitation in COPD is caused by the combination of parenchymal destruction (emphysema) and small airways disease (obstructive bronchiolitis), of which the relative presence varies from person to person [2].

According to estimates from the Global Burden of Disease Study, COPD was prevalent in more than 300 million people in 2013 [3]. The disease burden and its financial impact is predicted to increase, mainly due to population aging [4–6]. Several studies reported on the prevalence of COPD. In European adult populations over 40 years, the prevalence of COPD ranges between 15–20 % and is higher in men than in women [7–9]. Even though the prevalence of COPD is well known, only few studies examined its incidence rate in a prospective and standardized manner (supplementary Table 1S in the Online Resource provides an overview of studies which investigated the incidence of COPD).

While tobacco smoking is a major risk factor for COPD, only approximately 20 % of smokers develop the disease. More evidence is rising to suggest that other risk factors such as air pollution, respiratory infections, poor nutritional status, chronic asthma, impaired lung growth, poor socio-economic status and genetic factors are also important for disease development [10–12]. About 15–20 % of COPD cases are due to occupational exposures to pollutants at the workplace [9], and about 50 % of subjects who died from COPD in developing countries have been exposed to biomass smoke during lifetime [10]. These facts emphasize the need for action in order to reduce the impact of those risk factors on disease development. To this end, investigating the incidence of COPD is important, since it might shed light on new trends in the development and course of the disease, which in turn can lead to new insights and guidance for prevention and treatment. Therefore, the objective of this study is to investigate the prevalence and incidence of COPD by age, sex and smoking status in the participants of the Rotterdam Study, a large ongoing prospective population-based cohort study with 25 years of follow-up.

## Materials and methods

The present study was embedded within the Rotterdam Study, an ongoing prospective population-based cohort study that investigates the occurrence of chronic diseases and risk factors in elderly. The objective and methods of this cohort have been published previously [13, 14]. Briefly, the Rotterdam Study (RS) includes approximately 15,000 participants aged ≥45 years, living in Ommoord, a well-defined suburb of the city of Rotterdam, the Netherlands, and encompasses three cohorts: RS I, RS II and RS III. Baseline data were collected between 1989 and 1992 in RS I (c 7983), between 2000 and 2003 in RS II (*n* = 3011) and between 2006 and 2009 in RS III (*n* = 3932); thereafter cross-sectional surveys and examinations have been conducted every 4–5 years. Participants were initially interviewed at home for information on their health status. This was followed by an extensive set of examinations performed at a specially built research facility in the study district. Trained research assistants collected information from medical records of the general practitioners (GPs), nursing homes and hospitals. The study was approved by the medical ethics committee of Erasmus Medical Center, Rotterdam. All participants gave their written informed consent and permission to retrieve information from treating physicians.

### COPD diagnosis

COPD was diagnosed based on an obstructive pre-bronchodilator spirometry (FEV_1_/FVC < 0.70) according to the GOLD guidelines [2, 15, 16]. We also diagnosed COPD according to the lower level of normal (LLN) instead of GOLD as a sensitivity analysis as proposed by Hankinson et al. [17]. Spirometry was performed by trained paramedical personnel according to the ATS/ERS guidelines, using a portable spirometer (SpiroPro; Erich Jaeger GmbH; Hoechberg, Germany) from 2002 to 2008, and using a Master Screen^®^ PFT Pro (Care Fusion, the Netherlands) since 2009. Spirometry results which did not meet ATS/ERS criteria for acceptability were classified as not interpretable [18, 19]. Reversibility tests were not performed.

Within the Rotterdam study, pre-bronchodilator spirometry was performed in 8411 participants. In 7188 subjects, the spirometry met ATS/ERS criteria and was thus interpretable. In absence of an interpretable study-acquired spirometry, the medical records including letters from specialists and the electronic GP files were reviewed of all patients who regularly used medication for obstructive lung disease (Anatomical Therapeutic Chemical Classification codes: R03). Drug use was exclusively used for potential case finding; each such potential case was subsequently validated through careful evaluation of all medical records, hospitalizations and specialist letters and only included if a clear and well-founded diagnosis of COPD was retained. Cases were then defined as having physician diagnosed COPD based on clinical presentation and obstructive lung function measured by the GP or respiratory physician.

The incident date was defined as the date of the first obstructive lung function examination, the date of COPD diagnosis in the medical records or the date of first prescription of the COPD medication (in those with established COPD), whichever came first.

Prevalent cases were defined as having COPD at inclusion. Incident cases were defined as participants who acquired COPD during follow-up. For incident COPD cases, follow-up time was defined as the time period between the start of the study and the diagnosis of COPD, lost to follow up, death, or the last visit to the study centre (December, 2014).

### Statistical analysis

Statistical analysis was performed using SPSS statistical software (SPSS for Windows, version 21; SPSS; Chicago, IL), R (Foundation for Statistical Computing, Vienna, Austria) and Microsoft Excel (version 2010). For the statistical analyses, patients without informed consent for follow-up were excluded. The prevalence of COPD (%) was calculated by dividing the total number of COPD cases at baseline (prevalent cases) by the total number of participants included. The prevalence at the end of follow-up (%) was calculated as the total number of COPD cases at the end of this study divided by the total number of participants included. For the analysis of the incidence rate of COPD, patients with prevalent COPD at baseline were excluded. Median follow up time was estimated using the reverse Kaplan–Meier method (also called Kaplan–Meier estimate of potential follow-up method). Incidence rates were calculated by dividing the number of incident cases by the total number of person years of subjects at risk and are presented per 1000 person years. The 95 % Confidence Intervals (CI) were calculated using a Poisson distribution. Incidence rates were stratified for sex, age, and self-reported smoking behaviour at baseline. To study age-specific incidence rates, follow-up time was divided by five-year age intervals as described before [18]. Subjects contributed to the subsequent age intervals until they developed COPD, were lost to follow up, died, or reached the end of study (December, 2014). Smoking behaviour was categorised as current, former and never.

## Results

**Fig. 1:**
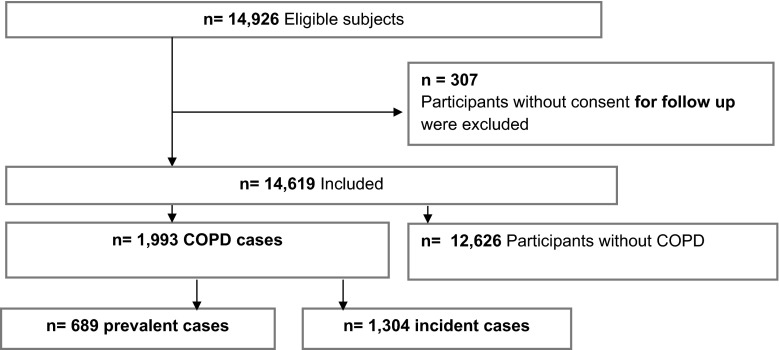
Flow chart of participants in the study

In this cohort of 14,619 participants with informed consent for follow-up, a total of 1993 individuals (56.5 % males) were identified as having COPD and 12,626 participants (38.8 % males) did not have COPD. Physician diagnosed asthma patients (*n* = 460) were excluded from the COPD cases, but were controls, as they were at risk to develop COPD. In addition, 54 asthma cases were added to the COPD group since they developed COPD during follow-up. A total of 311 of the 460 (68 %) asthma cases also performed an interpretable (pre-bronchodilator) spirometry within the Rotterdam Study of whom 60 had an obstructive lung function examination (FEV_1_/FVC < 0.7). A total of 689 COPD subjects were identified as having prevalent COPD at baseline and 1304 COPD cases were incident (Fig. 1). The median follow-up time was 10.7 years (with a maximum follow-up time up to 25 years) and mean age at baseline was 65.8 ± 10.4 years.

Regarding the smoking status, 21.7 % of the study participants were current smokers, 41.7 % former smokers and 34.2 % never smokers (Table 1). In ever smokers, 17.8 % (1663/9169) had COPD (including incident and prevalent cases), whereas in never smokers the prevalence of COPD was 6.4 % (318/4997). In men, 17.3 % (*n* = 1042/6024) were never smokers, compared to 46.0 % (*n* = 3955/8595) never smoking women. The proportion of COPD female cases without a smoking history was 27.2 % (236/867), while the proportion of never smokers among COPD male cases was 7.3 % (82/1126). Amongst the incident COPD patients who never smoked, questionnaire information on passive smoking was available in 170 patients. The proportion of passive smoking in these patients was 51.2 % (*n* = 87) and amongst these passive smokers, the majority were female (*n* = 67; 77 %).

**Table 1:** Baseline characteristics of the study population (*n* = 14,619)

Characteristics	Total *n* = 14,619	COPD cases *n* = 1993	Non-cases *n* = 12,626
Age (years) at baseline	65.8 (10.4)	64.8 (8.5)	65.9 (10.6)
Gender *n* (%)			
Males	6024 (41.2)	1126 (56.5)	4898 (38.8)
Females	8595 (58.8)	867 (43.5)	7728 (61.2)
Genetic ethnicity *n* (%)			
Central European	11,617 (98.0)	1638 (98.9)	9979 (97.9)
Asian	145 (1.2)	14 (0.8)	131 (1.3)
African	69 (0.6)	2 (0.1)	67 (0.7)
Admixed	21 (0.2)	3 (0.2)	18 (0.2)
Missing genetic data *n*	2767	336	2431
Smoking at baseline *n* (%)			
Current smoker	3078 (21.7)	800 (41.0)	2278 (18.6)
Former smoker	6091 (43.0)	831 (42.6)	5260 (43.1)
Never smoker	4997 (35.3)	318 (16.3)	4679 (38.3)
Missing *n*	453	44	409
Pack years of smoking mean (SD)			
Current smoker	30.3 (21.3)	34.6 (19.8)	28.7 (21.5)
Former smoker	22.0 (23.8)	33.6 (28.5)	20.2 (22.5)
Missing *n*	770	77	693
Anthropometry mean (SD)			
Weight (Kg)	76.0 (13.9)	76.2 (13.4)	76.0 (14.0)
Height (cm)	168.0 (9.6)	170.5 (9.4)	167.6 (9.5)
BMI	26.9 (4.1)	26.1 (3.9)	27.0 (4.1)
Missing *n*	1559	143	1416
Blood pressure mean (SD)			
Systolic blood pressure (mmHg)	139.6 (36.3)	138.1 (32.4)	139.8 (36.9)
Diastolic blood pressure (mmHg)	78.5 (33.7)	77.7 (29.1)	78.6 (34.4)
Missing *n*	1392	131	1261

Data are presented as *n* (% of valid total) or Mean ± standard deviation (SD)

The prevalence of COPD at baseline in the Rotterdam Study was 4.7 % (689/14,619) and the prevalence at the end of follow-up was 13.6 % (1993/14,619). The overall incidence rate (IR) of COPD was 8.9/1000 person-years (PY) (95 % CI 8.4–9.4/1000 PY). For the sensitivity analysis using LLN instead of the GOLD classification method, the overall incidence rate was 5.5/1000 PY (95 % CI 5.2–5.9) (See Table 2S in the online resource for detailed information on the prevalence and incidence data according to different classification methods; GOLD and LLN).

Subgroup analysis of the spirometry data based on GOLD (*n* = 7153) versus medical record data (*n* = 7466) was also performed. The prevalence of COPD was 5.3 versus 4.2 %, respectively. The incidence rate of COPD was 11.7/1000PY (95 % CI 10.9–12.4) versus 5.8/1000PY (95 % CI 5.3–6.4), respectively (Table 2S). Additional information is provided on severity and respiratory complaints in the spirometry group in online Table 3S.

**Fig. 2:**
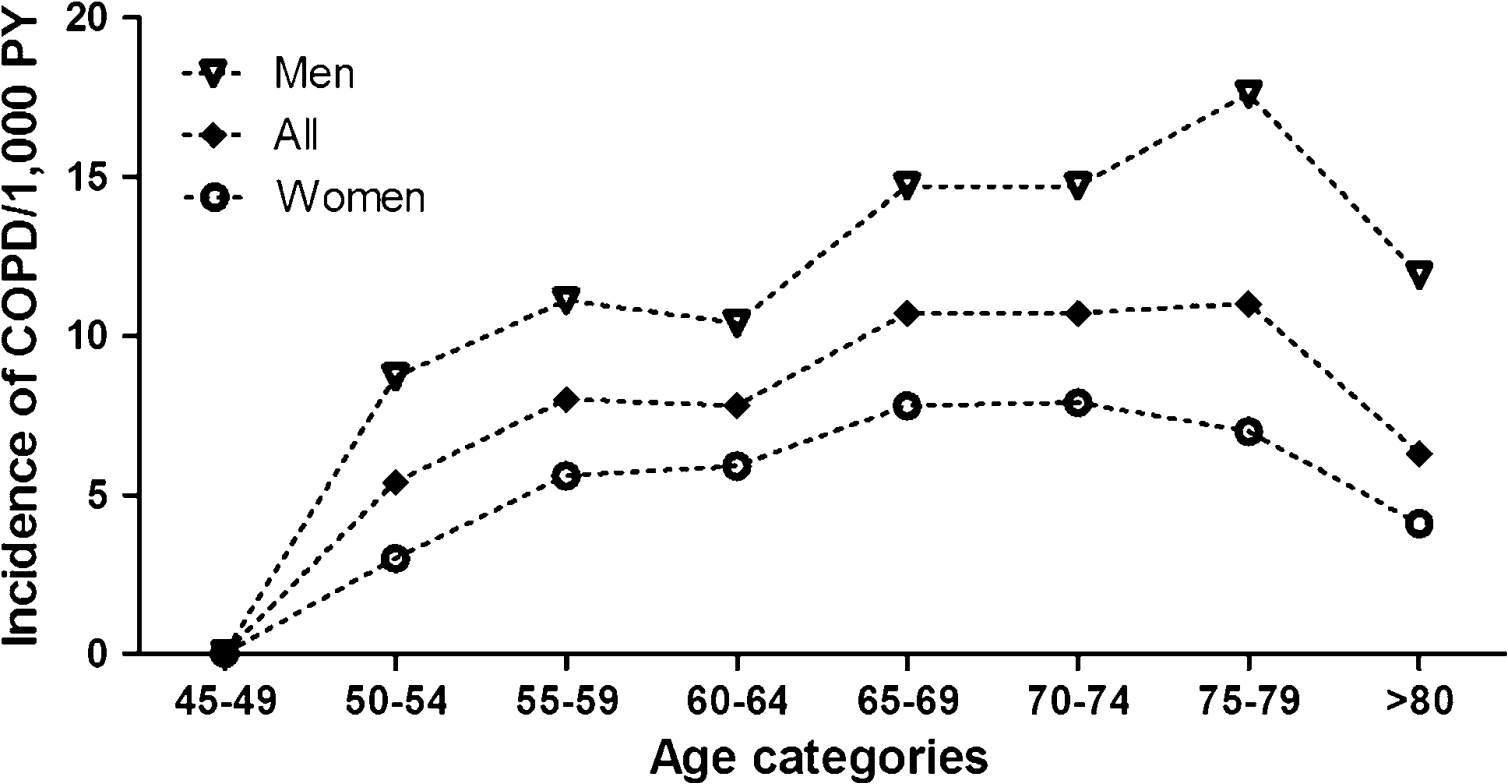
Age-specific incidence of COPD by sex

**Fig. 3:**
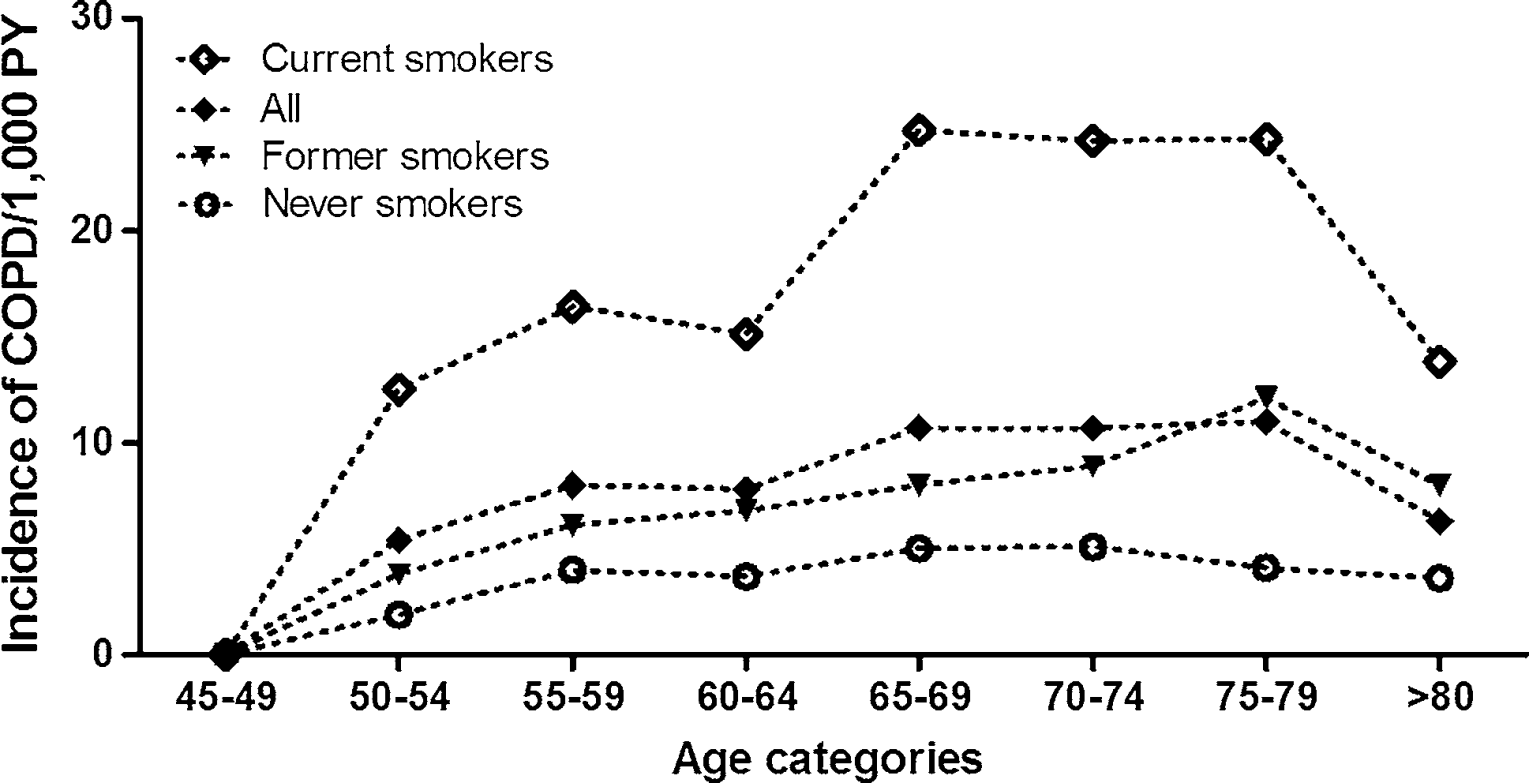
Age specific incidence of COPD by smoking behaviour

**Fig. 4:**
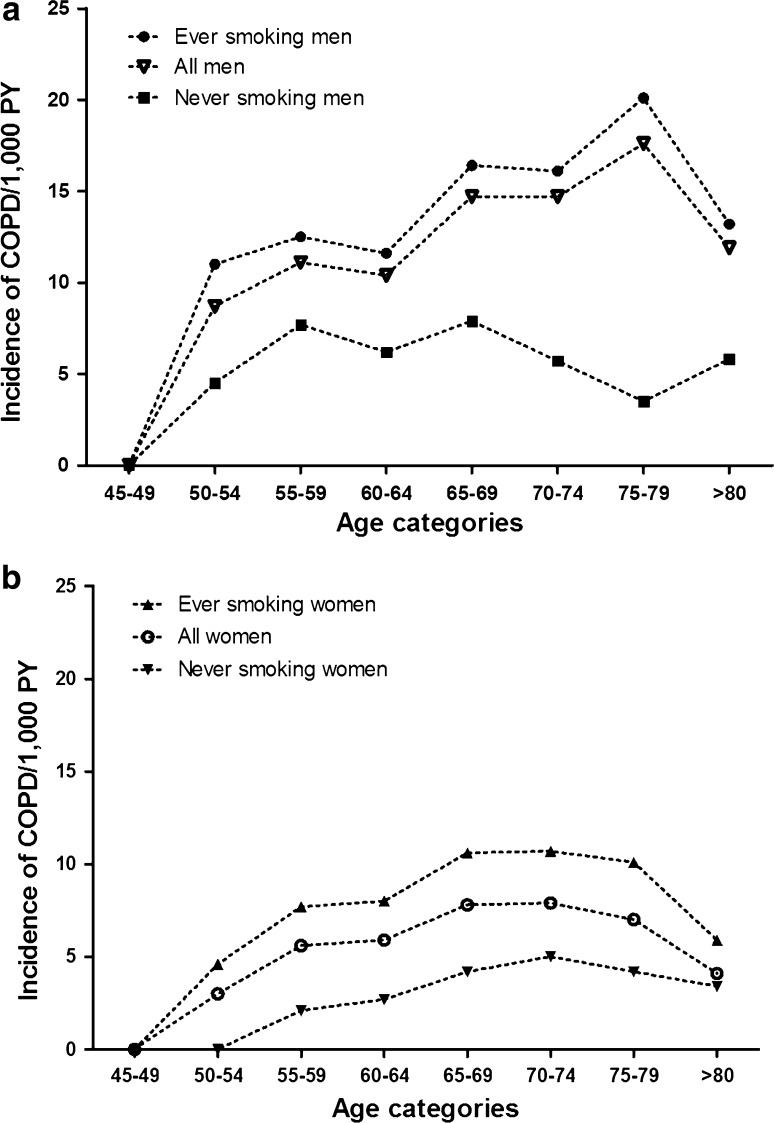
Age-specific incidence of COPD by sex (**a** men and **b** women) and smoking behaviour

The overall IR was higher in men (13.3/1000 PY, 95 % CI 12.4–14.3) than in women (6.1/1000 PY, 95 % CI 5.6–6.6); age specific IR ranged between 8.7 and 17.6/1000 PY in males and 3.0–7.9/1000 PY in females. The incidence of COPD increased from the age of 45 in both sexes to the age of 80 in men and 75 in women (Fig. 2). The IR was higher in current and former smokers than in never smokers (19.7/1000 PY, 95 % CI 18.1–21.4 in current smokers, 8.3/1000 PY, 95 % CI 7.6–9.1 in former smokers and 4.1/1000 PY, 95 % CI 3.6–4.7, in never smokers). The IR of COPD in smoking men was 15.0/1000 PY (95 % CI 13.9–16.2) compared to 8.6/1000 PY (95 % CI 7.8–9.5) in smoking women. The age-specific IR of COPD in ever smokers ranged between 7.3 and 15.3/1000 PY. The IR was 6.0/1000 PY (95 % CI 4.6–7.8) in never smoking men and 3.7/1,000 PY (95 % CI 3.1–4.3) in never smoking women. The age-specific incidence of COPD in never smokers increased by age, but to a lesser extent than the incidence of COPD in ever smokers (Fig. 3). After stratification by sex and smoking history, the age-specific incidence in never smoking women showed the same pattern (Fig. 4).

## Discussion

In this large prospective cohort study, the baseline prevalence of COPD was 4.7 % and the prevalence at the end of follow-up was 13.6 %. The overall incidence rate of COPD was approximately 9/1000 PY. This rate increased progressively with age, was higher in men than in women and higher in ever smokers compared to never smokers. Importantly, more than one in four female COPD subjects was a never smoker.

We previously published on the prevalence and incidence rates of COPD in the first Rotterdam Study (RS I; encompassing 7983 participants) over a follow-up period of 15.5 years (from 1989 till 2004) [18]. Here we report on the prevalence and incidence rates of COPD in all three RS cohorts (encompassing 14,926 participants) with an extended follow-up period of up to 25 years (from baseline till 2014). Comparing the results, the overall incidence rate in both studies showed high consistency (IR: 9.2/1000 PY [95 % CI; 8.5–10] in RS I versus 8.9/1000 PY [95 % CI; 8.4–9.4] in RS I, II and III combined). Given that both cohort studies used overlapping but different data sources (RS I versus RS I, II and III) and had different lengths of follow-up (15.5 versus 25 years), the consistency found highlights the reliability of the epidemiologic data.

Reviewing the literature, several studies reported on the prevalence of COPD. These prevalences varied widely and ranged from 0.2 % in Japan to 37 % in the USA and between 2.1 and 26.1 % in Europe [7, 8]. Bischoff et al. presented data on the prevalence of COPD in a dynamic general practice population aged 40 and older in the Netherlands using data from the Continuous Morbidity Registration [20]. Their prevalence (5.4 %) was in line with the one found in our study (4.7 %).

Numerous studies reported on the incidence of COPD but only few studies reported the incidence rate in large cohorts with long follow-up time taking the individual contribution to follow-up into account (see supplementary Table 1S). The reported measures on the incidence varied widely when reported in terms of rates per 1000 persons, ranging from 8.2 to 81.6 [21–23], while the incidence rates reported per person time units ranged from 2.6 to 9.2 per 1000 PY [18, 24–27]. This variation in incidence rates can be explained by variability in terms of the definition of COPD, research methods, source population and calendar time [7].

Overall, our study confirms that the incidence of COPD is higher in men than in women and in elderly (>75y) than in younger subjects. At our study centre, COPD was diagnosed based on an obstructive (pre-bronchodilator) spirometry. If an interpretable spirometry was not available, COPD was defined as a validated diagnosis made by the GP or the respiratory physician on the basis of clinical presentation and obstructive lung function. Therefore, not only symptomatic but (in contrast to the patients seen by the physician in the clinic) also asymptomatic or oligosymptomatic subjects with COPD were diagnosed in the RS. Since mild COPD cases rarely seek medical attention, the true incidence of COPD is frequently underestimated in clinical settings [23]. Studies that reported on physician diagnosed COPD showed lower IRs compared to the rate found in our epidemiologic study [24, 25, 28] (Table 1S). In our study, subgroup analysis of the spirometry data versus medical record data were also calculated. The incidence rates showed a similar pattern as compared to the literature and were 11.7/1000 PY using spirometry data versus 5.8/1000 PY using medical records data.

In this study, we classified COPD cases according to GOLD guidelines. Since ATS and ERS recently advocate the use of the Lower Limit of Normal (LLN), we also recalculated the incidence rate using LLN classification in the spirometry group instead of GOLD. The overall incidence rate after reclassifying the spirometry based COPD cases according to LLN was lower than the initial incidence rate using GOLD (5.5/1000PY versus 8.9/1,000 PY) which is in line with the literature [29]. This difference is ascribed to the fact that mild COPD cases as classified according to GOLD were considered as controls when LLN was used as a cut-off. Whether mild (asymptomatic) COPD should be classified as COPD is sometimes debated. However, Mannino et al. [30] demonstrated that subjects classified as “cases” using GOLD but as “normal” using LLN have a significantly higher risk of COPD-related hospitalization and mortality.

In our study, the age-specific incidence of COPD in never smokers increased by age, but to a lesser extent than the incidence of COPD in current and former smokers (Fig. 3). The detection of COPD cases in never smokers indicates that, besides tobacco smoking, other factors such as genetic susceptibility, impaired lung growth, respiratory infections and environmental exposures including occupational exposures and (outdoor and indoor) air pollution might contribute to the development of COPD [10–12]. Interestingly, in our study, approximately 27.2 % of all female COPD cases were never smokers, whereas this prevalence was much lower in men (7.3 %). This suggests that the contribution of environmental exposures other than active smoking leading to COPD seems more substantial in females than in males. Indeed, our data confirm that one of these environmental exposures, namely passive smoking, is higher in females than in males [31–33].

Likewise, more evidence is emerging on the increasing occurrence of COPD in non-smoking individuals, especially in females. Worldwide an estimated 25–45 % of patients with COPD never smoked [10]. Nevertheless, most randomized clinical trials (RCT) that examine the efficacy and safety of pharmacologic treatments for COPD, only include COPD patients with a history of cigarette smoking of at least 10 pack years [10].

The burden of COPD in never smokers is higher than previously believed [10, 11, 31, 34], therefore more research is needed to unravel the characteristics of non-smoking COPD in order to address the true burden, prognosis, clinical, radiographic and physiological features and treatment possibilities in this specific and neglected group.

The strengths of the Rotterdam study are the prospective, population-based design with a follow-up time of 25 years. In addition, measurements of the variables in this prospective cohort is done independently of the research question, which makes it less prone to information and selection bias.

A limitation is that spirometry measurements were introduced in the Rotterdam study in January 2002 and therefore measured in only 8411 participants (out of 9950 still alive). This could lead to an underestimation of asymptomatic COPD in the Rotterdam Study in participants without spirometry. A second limitation is that within the Rotterdam Study, as in most population-based cohort studies, reversibility tests were not performed, because the use of inhaled bronchodilators could interfere with other tests during the study visit. This could inflate the prevalence of COPD considerably [35, 36]. While some researchers state that the use of a bronchodilator is necessary to eliminate the variable airflow limitation in order to diagnose COPD [37], others suggest that bronchodilator responsiveness is anyway greatly variable and that more than 50 % of the patients who initially were classified as reversible would be reclassified, had they attended on a different occasion [38, 39]. The use of pre-bronchodilator spirometry implies that we cannot exclude the possibility of misclassification of some asthma patients as COPD patients. To minimize the risk of misclassification, we additionally identified and validated patients with physician-diagnosed asthma. However, we still acknowledge the use of pre-bronchodilatory test results as weakness because some unknown degree of inflation of COPD diagnoses might still be present.

In conclusion, the overall incidence rate of COPD in the Rotterdam Study was approximately 9/1000 PY, with a higher incidence in males and in smokers. The proportion of never smokers among COPD cases is substantial and higher in females than in males.

## Electronic supplementary material

Below is the link to the electronic supplementary material.

Supplementary material 1 (DOCX 55 kb)
