# Supplementary material for: Prevalence and incidence of COPD in smokers and non-smokers: the Rotterdam Study
Source: Eur J Epidemiol. 2016 Mar 5;31(8):785–92. doi: 10.1007/s10654-016-0132-z (PMC5005388; doi:10.1007/s10654-016-0132-z)
Supplement: Supplementary file 1 — Supplementary material 1 (DOCX 55 kb) [file 10654_2016_132_MOESM1_ESM.docx]

**Prevalence and incidence of COPD in smokers and non-smokers:**

**the Rotterdam Study**

Natalie Terzikhan, MSc^1,2^ , Katia M.C. Verhamme, PhD^6^, Albert Hofman, PhD^2^, Bruno H. Stricker, PhD^2,3,4^ , Guy G. Brusselle, PhD^1,2,5^ , Lies Lahousse, PhD^1,2^

**To the European Journal of Epidemiology**

**Correspondence:**
Bruno H Stricker, MMed PhD Department of Epidemiology, Erasmus MC – University Medical Center Rotterdam,
PO Box 2040, 3000 CA Rotterdam, the Netherlands
[b.stricker@erasmusmc.nl](mailto:b.stricker@erasmusmc.nl); phone 0031 10 70 44292; fax 0031 10 70 44657

**Table 1S Overview of studies which investigated the incidence of COPD**

| Reference | Study period | Source population | Database name | Country | Number  (cohort/cases) | Age range at entry (years) | COPD diagnosis | Incidence estimate |
| --- | --- | --- | --- | --- | --- | --- | --- | --- |
| *Diagnosis based studies* | |  |  |  |  |  |  |  |
| Crighton et al. 2015[[20](#_ENREF_20)] | **2002-2010** | Population based-claims database | Ontario health administrative databases. Four databased were linked | Canada | COPD cases:  57,159/Ontario population | ≥ 35 | “One COPD hospitalization and/or one COPD ambulatory case claim as indicated by the following codes: 491,492,496 or J41, J42, J43, J44” | Overall incidence:  8.2/1,000 persons  --- “In all age groups, incidence rates were higher among men than women, with the highest rate among men aged 65 or older (16.3 per 1,000 persons)” |
| Afonso et al. 2011[[23](#_ENREF_23)] | **2000-2007** | Population based-IPCI database | IPCI database | The Netherlands | COPD cases:  7,308 of which 1,713 had incident COPD | ≥40 | “Definite COPD: diagnosis by a specialist or a GP diagnosis confirmed by spirometry (GOLD definition of FEV1/FVC<70%)” | Overall incidence:  2.92/1,000 PY (95% CI 2.78-3.06).  ---  The incidence was higher in men than in women |
| Gershon et al. 2010[[21](#_ENREF_21)] | **1996, 2002 and 2007** | - Population based-claims database  - Longitudinal cohort study using population based health administrative data | - Ontario Health Insurance Plan database for physician claims  - Canadian institute for Health Information database for hospital admissions | Canada | n total:  5-6.4 million  COPD cases: 61,998 (1996)  52,158 (2002)  55,903 (2007) | ≥ 35 | “One or more physical billing claims and/or 1 or more hospital discharges of COPD as per the following codes: 490-492, 496 (ICD-9) or J41-44 (ICD -10)” | The age and sex standardized incidence /1,000 persons:  All ages:  - 11.8 ( in 1996)  - 8.9 (in 2002)  - 8.5 (in 2007)  No CI presented  ---  Incidence was higher in males than females and by increased age |
| Garcia Rodriguez et al. 2009[[24](#_ENREF_24)] | **1996** | Population based-GPRD | GPRD database, followed by a nested case control study | UK | n total: 808,513  Potential COPD cases: 2351 | 40-89 | COPD diagnosed in OXMIS and Read coding | Overall incidence:  2.6 /1,000 PY (95% CI 2.5-2.7)  ---  “Incidence was lowest in the age group 40-49 years and highest in the highest age group 80-89 years” |
| Nihlen et al. 2004[[27](#_ENREF_27)] | **1992 and 2000** | Postal questionnaires in 1992 and 2000 | NR | Sweden | n responders to both questionnaires: 4,280 | 28-67 | Self-reported physician diagnosed CBE/COPD | 8-year cumulative incidence of a physician's diagnosis of CBE/COPD 2.9% (95% CI 2.4-3.4)  ---  Corresponding to an estimated annual incidence of 0.36% |
| Mannino et al. 2002[[22](#_ENREF_22)] | **1971-2000** | Population based survey data of COPD at physician office, hospital and emergency department | NAMCS/NHAMCS | USA | 30,000 visits to physician’s office, outpatients department encounters and emergency department encounters | ≥ 25 | COPD as first stated diagnosis (ICD-9 code: 490-492, 496) | Incidence /1,000 persons at physician office and outpatient department visits:  - Overall: 45.0  - Over time 44.5-81.6 (1980-1998) 58.9-45.0 (1999-2000)    Incidence per 1,000 persons at emergency department visits:  - Overall: 8.72  - Over time: 67.6-84.9 (1992-1995), 72.7-87.2 (1997-2000) |
| *Spirometry based studies* | |  |  |  |  |  |  |  |
| Omori et al. 2011[[28](#_ENREF_28)] | **1994 and 2006** | Longitudinal study using health check-up data in males | Longitudinal study of the Japanese Red Cross Kumamoto Health Care Centre | Japan | n total: 913  COPD cases: 91 | 30-76 | COPD was diagnosed according to the GOLD guidelines using spirometry (fixed criteria and lower limit of normal criteria) | Mean annual incidence using:  - **Fixed criteria** (FEV_1_/FVC <0.70): 0.83%  **- LLN** (FEV_1_/FVC <LLN): 0.98% |
| Van Durme et al. 2009[[17](#_ENREF_17)] | **1990-2008** | Prospective cohort study, general population sample without COPD at baseline | The Rotterdam study | The Netherlands | n total: 7,983  COPD cases: 648 | ≥ 55 | COPD diagnosed according to GOLD guidelines using spirometry and discharge letters for spirometry | Incidence/1,000 PY:  Overall: 9.2 (95% CI 8.5-10)  ---  “Incidence was higher among men than in women and in smokers than in never smokers” |
| Kojima et al. 2007[[25](#_ENREF_25)] | **1997-2005** | Health check-up data in males and females | Large longitudinal study of the Toyota Regional Medical Centre | Japan | n total: 17,106  COPD cases: 466 | 25-74 | COPD diagnosed according to GOLD guidelines using spirometry (GOLD stage I and higher) | Incidence/1,000 PY by gender:  - Men: 8.1  - Women: 3.1  No CI reported  ---  “Incidence was higher in older age categories” |
| De Marco et al. 2007[[26](#_ENREF_26)] | **1991-1993 ECRHS I**  **1999-2002 ECRHS II** | Survey. Participants were invited by questionnaire. Of the responders, a random sample of 20% were invited for detailed clinical examination including spirometry | ECRHS | USA and Europe | COPD cases: 5002 | 20-44 | COPD was diagnosed using FEV_1_/FVC ratio <70% | Incidence/1,000 PY  - Overall: 2.8 (95% CI 2.3-3.3)  - Men: 3.2 (95% CI 2.5-4.1)  - Women: 2.4 (95% CI 1.8-3.1)  - By age categories (years) :  20-30: 1.5 (95% CI 1.0-2.3)  30-40: 2.6 (95% CI 1.9-3.4)  40-45: 4.7 (95% CI 3.6-6.1) |
| Lindberg et al. 2006[[29](#_ENREF_29)] | **1996 and 2003** | Population based cohort with survey related to respiratory symptoms and diseases and subgroup invited for functional interview and examination | OLIN study | Sweden | n total: 963 performed spirometry in both occasions  COPD cases:  91 (GOLD I and higher)  45 (GOLD II and higher) | 46-77 | COPD diagnosed according to GOLD guidelines using spirometry (grade I and higher) | 7-year cumulative incidence of COPD:  **- GOLD I and higher**: 11.0%  **- GOLD II and higher**: 4.9%  ---  Corresponding annual incidence rates (rates/1,000 persons/year):  **GOLD I and higher**:  - Overall: 16  - Smokers: 27  - Non-smokers: 11  **GOLD II and higher**:  - Overall: 7  - Smokers: 15  - Non-smokers: 2 |
| Geijer et al. 2006[[30](#_ENREF_30)] | **1998 and 2003** | Prospective cohort study in smoking males without current lung disease registered with a GP in Ijsselstein, a small town in The Netherlands,  All men registered with a GP in Ijsselstein were asked to participate via postal form related to smoking | Ijsselstein cohort study | The Netherlands | - 1998:  n total: 918  n spirometry: 702  - 2003:  n total: 436 | 40-65 | COPD was diagnosed according to GOLD guidelines using spirometry  ---  Mild COPD patients were included at baseline | Cumulative incidence after a mean follow-up of 5.2-year: 8.3% (95% CI 5.8 to 11.4)  ---  Mean annual incidence of 1.6% |
| Lokke et al. 2006[[31](#_ENREF_31)] | **1976-1978**  **1981-1983**  **1991-1994**  **2001-2003** | Prospective cohort study, general population sample without COPD at baseline | The Copenhagen City Heart Study | Denmark | - n total: 2,442 participated in the first and fourth follow up examinations only  - n total:2,022 participated in all four examinations | 30-60 | “COPD staging was done according to the criteria of the American Thoracic Society and the European Respiratory Society” | 25-year cumulative incidence:  - Moderate COPD 20.7%  - Severe 3.6%  ---  No apparent difference was found between men and women |
| Lindberg et al. 2005[[32](#_ENREF_32)] | **1986 and 1996** | Population based cohort with survey related to respiratory symptoms and diseases and subgroup invited for functional interview and examination. | OLIN study | Sweden | - 1986: 1506 interviewed and examined  - 1996: 1109 with adequate spirometry | 36-67 | COPD was defined according to the BTS and GOLD using spirometry | 10-year cumulative incidence:  **BTS**  - Overall: 8.2%  - Male: 9.0%  - Female: 7.5%  ---  **GOLD**  - Overall: 13.5%  - Male: 15.3%  - Female: 11.8%  ---  “Incidence in persistent smokers was higher compared to non-smokers” |
| Johoannessen et al. 2005[[33](#_ENREF_33)] | **1985**  **1987-1988**  **1996-1997 (spirometry)** | Postal questionnaire in 1985, a questionnaire in 1987-1988 and spirometry (1996-1997) | NR | Norway | n total: 908 | 18-74 | COPD was diagnosed according to GOLD guidelines using spirometry | Annual incidence: 7/1,000 PY  9- year cumulative incidence: 6% |
| Vestbo et al. 2002[34] | **1976-1978**  **1981-1983**  **1992-1994** | Prospective cohort study, general population sample without COPD at baseline | Copenhagen City Heart Study | Denmark | n total: maximal 14,223 COPD cases: not mentioned | ≥ 20 | Spirometry | 5- and 15-year cumulative incidence in smokers:  - 5 years: 13.2%  - 15 years: 20.5%  ---  5- and 15-year cumulative incidence in smokers without respiratory symptoms:  - 5 years: 11.6%  - 15 years: 18.5% |

BTS: British thoracic society; CBE: chronic bronchitis and/or emphysema; CI: Confidence interval; COPD: Chronic obstructive pulmonary disease; ECRHS: European community respiratory health survey; FEV_1_: Forced expiratory volume in one second; FVC: Forced vital capacity; GOLD: Global initiative for chronic obstructive lung disease; GP: General practitioner; GPRD: General proactive research database; ICD: International classification of diseases; IPCI: Integrated primary care information; LLN: Lower level of normal; N: number; NAMCS: National ambulatory medical care survey; NHAMCS: National hospital ambulatory medical care survey; NR: Not reported; OLIN: The obstructive lung disease in northern Sweden; OXMIS: Oxford medical information system; PY: Person years; UK: United kingdom; USA: United States of America

**Table 2S Prevalence and incidence data according to different classification methods in the total cohort and in the sub-groups (spirometry *versus* medical charts group)**

|  | Spirometry data | Medical records data | Combined data |
| --- | --- | --- | --- |
|  | N=7,153 | N=7,466 | N=14,619 |
| GOLD |  |  |  |
| *Prevalence* | 5.3% | 4.2% | 4.7% |
| *Incidence* | 11.7/1,000PY | 5.8/1,000PY | 8.9/1,000PY |
| LLN |  |  |  |
| *Prevalence* | 3.4% | 4.2% | 3.8% |
| *Incidence* | 5.2/1,000PY | 5.8/1,000PY | 5.5/1,000PY |

**Table 3S Spirometric severity and respiratory symptoms of COPD cases based on the most recent study-acquired spirometry and most recent questionnaire round***

|  |  | COPD cases | Prevalent COPD | Incident COPD |
| --- | --- | --- | --- | --- |
| Total |  | 1,183 (100) | 356 (100) | 827 (100) |
| Degree of airflow limitation | |  |  |  |
|  | ***Mild*** | 594 (50.3) | 155 (43.5) | 439 (53.1) |
|  | ***Moderate-severe*** | 589 (49.7) | 201 (56.5) | 388 (46.9) |
| Respiratory symptoms | |  |  |  |
|  | ***Dyspnea (yes)*** | 559 (47.3) | 202 (56.7) | 467 (56.5) |
|  | ***Dyspnea (no)*** | 616 (52.1) | 149 (41.9) | 357 (43.2) |
|  | *Missing* | 8 (0.7) | 5 (1.4) | 3 (0.4) |
|  | ***Cough & sputum (yes)*** | 316 (26.7) | 80 (22.5) | 236 (28.5) |
|  | ***Cough & sputum (no)*** | 860 (72.7) | 272 (76.4) | 588 (71.1) |
|  | *Missing* | 7 (0.6) | 4 (1.1) | 3 (0.4) |

*Figures are presented as n (%)
